# Risk Factors for Postoperative Complications in Hallux Valgus Surgery: A Retrospective Cohort Study

**DOI:** 10.3390/jcm15145709

**Published:** 2026-07-21

**Authors:** Isabel Buendía-Ayala, Josefa Buendía-Ayala, Lucille Ridgell, Camila Miño, José Francisco López-Gil, Pedro Juan Tarraga-López, Mateo Amando López-Cara

**Affiliations:** 1 Department of Orthopedic Surgery and Traumatology, Hospital General Universitario Reina Sofia, 30003 Murcia, Spain; buendiaayala@gmail.com; 2 University of Castilla-La Mancha, 02071 Albacete, Spain; pedrojuan.tarraga@uclm.es; 3 University of Murcia, 30071 Murcia, Spain; josefa.buendia@um.es; 4 Carver University, Orlando, FL 32804, USA; lucilleridgell@gmail.com; 5 Universidad Internacional para el Desarrollo (UNINDE), 06011 Badajoz, Spain; 6 School of Medicine, Universidad Espíritu Santo, Samborondón 0901952, Ecuador; 7 Faculty of Health Sciences, Universidad Autónoma de Chile, Temuco 4780000, Chile; 8 Castilla-La Mancha Health Service (SESCAM), 45071 Toledo, Spain; mateoamando@yahoo.es

**Keywords:** hallux valgus, postoperative complications, risk factors, diabetes mellitus, smoking, foot surgery, osteotomy

## Abstract

**Background/Objectives**: Hallux valgus is a common forefoot deformity whose surgical correction generally yields favorable functional outcomes. However, postoperative complications remain a clinically relevant concern, and the contribution of preoperative risk factors has not been comprehensively characterized in the context of the Spanish population. This study aimed to analyze the relationship between preoperative risk factors and the occurrence of complications after hallux valgus surgery, and to assess whether their accumulation follows a dose–response pattern of increased postoperative risk. **Methods**: A retrospective observational study was conducted in 123 consecutive patients who underwent hallux valgus surgery between 1 January 2017 and 31 December 2018, with a mean follow-up of 12 months. Variables analyzed included age, sex, deformity severity, active smoking, diabetes mellitus, and antiplatelet or anticoagulant therapy. An accumulated risk factor index based on three clinical risk factors was constructed. Statistical analysis included descriptive statistics, the Cochran–Armitage trend test, and multivariate logistic regression. **Results**: The mean age was 57.4 years; 82.1% were female. Active smokers accounted for 31.7%, 14.6% had diabetes mellitus, and 13.0% were receiving antithrombotic therapy. The overall complication rate was 20.3%, comprising recurrence (12.2%), chronic residual pain (6.5%), joint stiffness (4.1%), and infection (4.1%). Complication frequency increased progressively from 15.9% in patients without risk factors to 50.0% in those with three concurrent factors (*p*-trend = 0.038). In multivariate analysis, male sex (odds ratio [OR] 5.39; 95% confidence interval [CI] 1.41–21.19; *p* = 0.014) and diabetes mellitus (OR 8.37; 95% CI 1.42–63.60; *p* = 0.026) were independently associated with a higher risk of complications. **Conclusions**: Hallux valgus surgery generally has a favorable prognosis; however, the presence of preoperative risk factors significantly influences postoperative risk. Diabetes mellitus and the accumulation of risk factors identify patients who could benefit from more rigorous preoperative evaluation, metabolic optimization, and closer postoperative follow-up.

## 1. Introduction

Hallux valgus is the most prevalent structural deformity of the forefoot in the adult population, affecting approximately 23% of adults and up to 35% of individuals over the age of 65 years [1,2]. It is characterized by progressive lateral deviation of the hallux with concurrent medial displacement of the first metatarsal head, subluxation of the metatarsophalangeal joint, and functional disruption of the sesamoid complex [3,4,5]. The condition predominantly affects women, with a female-to-male ratio ranging from 2:1 to 4:1 across epidemiological series [6], and its pathogenesis is multifactorial, involving an interplay of genetic predisposition, ligamentous laxity, altered first-ray geometry, and external factors such as constrictive or high-heeled footwear [7]. Beyond these well-established biomechanical and genetic contributors, recent Mendelian randomization evidence has also proposed thyroid dysfunction as a potential systemic risk factor for hallux valgus, illustrating the growing interest in identifying modifiable and non-modifiable patient-level determinants of this deformity [8].

The clinical burden of hallux valgus is substantial. Patients frequently report significant metatarsophalangeal pain, functional limitation, impaired gait and balance, footwear intolerance, and reduced health-related quality of life [1,6]. These functional consequences are particularly relevant in older patients, in whom the deformity may contribute to instability and risk of falls. Conservative management, including custom orthoses, shoe modification, and physiotherapy, may provide symptomatic relief in mild cases but does not correct the underlying structural deformity [3,9]. When conservative measures fail to provide adequate relief after a minimum of three to six months, or when the deformity is structurally progressive or associated with transfer lesions, surgical correction is indicated.

Surgical technique selection follows established severity-based criteria (distal, diaphyseal, or proximal osteotomy, with adjunctive Akin osteotomy as needed) [9,10,11]. Postoperative outcomes, however, are not determined solely by the surgical technique but are also significantly influenced by the patient’s systemic and metabolic condition, as detailed below.

Although surgical outcomes are generally favorable, postoperative complications remain a non-negligible concern. Reported complication rates in contemporary series range broadly from 10% to 35%, encompassing deformity recurrence, chronic residual pain, joint stiffness, wound healing disorders, surgical site infection, hardware intolerance, and transfer metatarsalgia [9,10,11,12,13,14]. This variability reflects heterogeneity in patient selection, surgical technique, follow-up duration, and complication definition across studies. Importantly, the systematic contribution of preoperative risk factors to this risk burden has received limited focused attention in the hallux valgus literature. A recent bibliometric analysis of the global hallux valgus literature similarly identified patient-level risk stratification and postoperative outcomes as emerging, comparatively under-explored research hotspots [15].

Risk factors such as diabetes mellitus, active smoking, and anticoagulation therapy have well-established deleterious effects on tissue healing, immune defense, and bone metabolism in the orthopedic context [16,17,18,19]. Diabetes mellitus impairs wound healing through microangiopathic changes, immune dysfunction, and altered collagen synthesis [16,18,19]. Smoking reduces tissue oxygen tension, suppresses fibroblast activity, and is independently associated with increased rates of wound dehiscence, infection, and delayed union in foot and ankle surgery [17,20]. Antiplatelet and anticoagulant therapies are associated with increased perioperative hemorrhagic risk and potential interference with bone healing, although the evidence in the foot surgery context remains limited.

Beyond individual risk factors, the simultaneous presence of multiple risk factors may confer a risk that exceeds the sum of its individual components. Cumulative risk factor burden tools, such as the Charlson Comorbidity Index, have demonstrated that the co-occurrence of systemic conditions amplifies perioperative and postoperative risk across multiple surgical specialties [21]. Whether this cumulative effect is observable and quantifiable in hallux valgus surgery has not been directly tested in the available literature.

The aim of the present study was to analyze the relationship between preoperative risk factors (i.e., specifically diabetes mellitus, active smoking, and antithrombotic therapy) and the occurrence of postoperative complications following hallux valgus surgery in a real-world clinical cohort. A secondary objective was to assess whether the accumulation of these risk factors preoperatively follows a dose–response pattern of increasing postoperative risk, providing a clinically applicable risk stratification framework.

## 2. Materials and Methods

### 2.1. Study Design and Patient Selection

A retrospective observational study was conducted in consecutive patients who underwent elective hallux valgus surgery at the Hospital General Universitario Rafael Mendez (Lorca, Murcia, Spain) between 1 January 2017 and 31 December 2018. The Hospital General Universitario Rafael Mendez is a public secondary-level hospital serving the health area of Lorca, with a catchment population of approximately 120,000 inhabitants. The Orthopedics and Traumatology unit performs approximately 80–100 elective foot and ankle procedures annually. The study was approved by the Research Ethics Committee of Health Area III of the Murcian Health Service (Comité de Investigación, Area III, Servicio Murciano de Salud; approval dated 15 November 2019), and was conducted in accordance with the Declaration of Helsinki. All patients provided written informed consent to the processing of their anonymized clinical data. Protocol no. 2029/1115. Baseline, exposure, and outcome data were collected retrospectively from electronic clinical records and from documentation recorded at scheduled follow-up visits using structured questionnaires.

Inclusion criteria were: age between 18 and 85 years; clinical and radiological diagnosis of symptomatic hallux valgus with a formal surgical indication after failure of conservative treatment for a minimum of three months; and availability of complete preoperative and postoperative medical records including radiological imaging. Exclusion criteria included: previous ipsilateral forefoot surgery; preoperative diagnosis of peripheral neuropathy or active diabetic foot; inflammatory arthropathy (rheumatoid arthritis, gout, or psoriatic arthritis); concurrent metatarsalgia requiring simultaneous lesser toe surgery; and bilateral simultaneous procedure. A total of 123 patients met the eligibility criteria and were included in the final analysis.

Preoperative data collected included: age, sex, body mass index (BMI), smoking status (active smoker defined as current use at the time of surgery), diagnosis of diabetes mellitus (type 1 or type 2, confirmed by clinical record and antidiabetic treatment), and use of antiplatelet or anticoagulant therapy (antithrombotic therapy). Antithrombotic therapy was analyzed as a proxy marker for underlying cardiovascular or thromboembolic indications (e.g., atrial fibrillation, coronary artery disease, prior venous thromboembolism) rather than as an independent disease entity. These three variables were selected because they were the only exposures consistently and reliably documented in structured, extractable format in the electronic clinical records for the 2017–2018 study period; a complete comorbidity and medication profile was not available for retrospective reconstruction. All three risk factors were treated as dichotomous variables for the construction of the risk factor index. Radiological assessment was performed on standardized weight-bearing anteroposterior and lateral foot radiographs. Deformity severity was classified using the Coughlin and Mann criteria based on the hallux valgus angle (HVA) and the first-second intermetatarsal angle: mild (HVA < 25 degrees), moderate (HVA 25–40 degrees), and severe (HVA > 40 degrees) [6,22].

Preoperative functional status was assessed using two validated scales: the Visual Analogue Scale for pain (VAS, range 0–10, where 0 = no pain and 10 = maximum pain), and the American Orthopaedic Foot and Ankle Society Hallux Metatarsophalangeal-Interphalangeal (AOFAS-MI) scale (range 0–100, where 100 = perfect function) [23]. Surgical technique was determined by deformity severity. Patients with mild hallux valgus (HVA < 25 degrees; 22.8%) underwent Chevron distal osteotomy; those with moderate (HVA 25–40 degrees; 59.3%) or severe (HVA > 40 degrees; 17.9%) deformity underwent Scarf diaphyseal osteotomy. In all cases, a complementary Akin proximal phalanx osteotomy was performed to correct residual hallux valgus interphalangeus. All procedures were performed under locoregional anesthesia and tourniquet, with internal fixation using cortical screws. Postoperative management was standardized: partial weight-bearing in a rigid-soled postoperative shoe was allowed from day one, with progressive transition to normal footwear at six to eight weeks according to radiological consolidation.

An accumulated risk factor index was constructed based on the number of preoperative risk factors present (active smoking, diabetes mellitus, and antithrombotic therapy), yielding a score ranging from 0 to 3. This categorical composite variable was designed to capture the cumulative systemic burden at the individual patient level, following the conceptual framework of cumulative risk factor burden tools [21]. The mean follow-up period was 12 months (range: 10–18 months).

### 2.2. Outcome Assessment and Follow-Up Protocol

Clinical and radiological follow-up visits were scheduled at 6 weeks, 3 months, 6 months, and 12 months postoperatively. At each visit, VAS and AOFAS-MI scores were recorded, radiological consolidation was assessed, and a systematic complication review was performed using a structured questionnaire. Postoperative complications were defined as any adverse event requiring additional clinical management or surgical revision, and were coded into four predefined categories: (1) deformity recurrence, defined as HVA > 20 degrees on postoperative radiograph at six months or later with recurrent symptomatic deformity; (2) chronic residual pain, defined as VAS ≥ 4 persisting beyond three months postoperatively despite conservative management; (3) joint stiffness, defined as passive range of motion of the first metatarsophalangeal joint below 30 degrees of dorsiflexion at six months; and (4) surgical site infection, classified as superficial (involving skin and subcutaneous tissue only) or deep (extending to fascia or bone), according to the Centers for Disease Control and Prevention criteria.

### 2.3. Statistical Analysis

Quantitative variables were described using means and standard deviations (SDs); qualitative variables were expressed as absolute frequencies and percentages. Inter-group comparisons for categorical variables were performed using the chi-squared test or Fisher’s exact test as appropriate. For continuous variables, the Student *t*-test or Mann–Whitney U test was applied based on normality assessed by the Shapiro–Wilk test.

The Cochran–Armitage trend test was used to evaluate the dose–response relationship between the accumulated risk factor index and the overall complication rate. Inter-surgeon variability in complication rates was assessed using Fisher’s exact test.

A multivariate binary logistic regression model was fitted with the occurrence of any postoperative complication as the dependent variable. Given the limited number of events (*n* = 25), two nested models were pre-specified. The primary model included only male sex and diabetes mellitus, the two predictors with bivariate significance and consistent prior evidence as established risk factors (12.5 events per variable). A secondary, sensitivity model additionally included age (continuous), sex, active smoking, diabetes mellitus, antithrombotic therapy, and severity of hallux valgus (ordinal: mild, moderate, severe) to explore the direction and relative magnitude of associations across the full candidate covariate set. This model is exploratory rather than confirmatory given its lower events-per-variable ratio (3.6). Model selection was guided by clinical plausibility and collinearity assessment (variance inflation factor <5 for all variables). Results are reported as odds ratios (ORs) with 95% confidence intervals (CIs). Model calibration was assessed using the Hosmer–Lemeshow goodness-of-fit test. Statistical significance was set at two-tailed *p* < 0.05. All analyses were performed using RStudio (version 4.3; Posit PBC, Boston, MA, USA).

## 3. Results

### 3.1. Sample Characteristics

A total of 123 patients were included. The mean age was 57.4 years (SD 12.3; range: 22–81 years), and 101 patients (82.1%) were female. Thirty-nine patients (31.7%) were active smokers at the time of surgery, 18 (14.6%) had a confirmed diagnosis of diabetes mellitus (15 type 2, 3 type 1), and 16 (13.0%) were receiving antiplatelet or anticoagulant therapy (10 antiplatelet, 6 anticoagulant). The mean BMI was 27.3 kg/m^2^ (SD 4.1). Regarding deformity severity, 28 patients (22.8%) had mild, 73 (59.3%) moderate, and 22 (17.9%) severe hallux valgus. Two surgeons performed all procedures: Surgeon A (*n* = 72; 58.5%) and Surgeon B (*n* = 51; 41.5%). Detailed baseline characteristics are presented in Table 1.

### 3.2. Functional Outcomes

At 12 months, significant functional improvements were observed in all patients who completed follow-up. The mean VAS pain score decreased from 7.4 (SD 1.1) preoperatively to 1.2 (SD 0.7) postoperatively, representing a mean reduction of 6.2 points (83.8%; 95% CI 6.0–6.4; *p* < 0.001). The mean AOFAS-MI score improved from 56.0 (SD 7.5) preoperatively to 91.0 (SD 4.7) postoperatively, with a mean gain of 35.0 points (62.5%; 95% CI 33.5–36.6; *p* < 0.001). These improvements were observed across all subgroups defined by deformity severity and were maintained at the final follow-up visit.

### 3.3. Postoperative Complications

The overall complication rate was 20.3% (*n* = 25 patients). Deformity recurrence was the most frequent complication, observed in 15 patients (12.2%). Chronic residual pain was recorded in 8 patients (6.5%), joint stiffness in 5 (4.1%), and surgical site infection in 5 (4.1%). All five infections were superficial and resolved with oral antibiotic therapy without the need for surgical debridement or reoperation. Eight patients (6.5%) experienced two simultaneous complications. No statistically significant difference in complication rates was observed between the two operating surgeons (19.4% vs. 21.6%; *p* = 0.72, Fisher’s exact test), confirming that the results are not attributable to surgeon-level variability.

### 3.4. Complication Rate Analysis by Individual Risk Factor

Analysis of complication rates by individual risk factors showed a consistent pattern of increased risk in the presence of any risk factor (Table 2). Patients with active smoking had a complication rate of 28.2% (11/39) compared with 15.5% (13/84) in nonsmokers, though this difference did not reach statistical significance (*p* = 0.12). Patients with diabetes mellitus had a complication rate of 38.9% (7/18) versus 17.1% (18/105) in those without diabetes (*p* = 0.049). Patients receiving antithrombotic therapy had a complication rate of 31.3% (5/16) versus 18.7% (20/107) in those not receiving such therapy (*p* = 0.31). These unadjusted comparisons should be interpreted with caution given the small subgroup sizes and the confounding present at the bivariate level.

### 3.5. Analysis of Risk Factor Burden and Complication Rate

**Figure 1:**
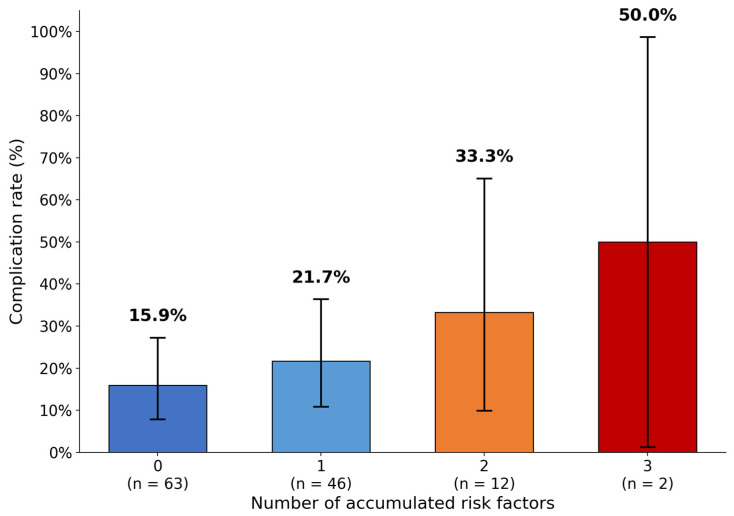
Complication rate (%) according to the number of accumulated risk factors (0–3), with error bars representing exact (Clopper–Pearson) 95% confidence intervals. Cochran–Armitage trend test: *p*-trend = 0.038.

When analyzed according to the accumulated risk factor index, a progressive and statistically significant increase in complication rate was observed: 15.9% (10/63) in patients without any risk factor, 21.7% (10/46) with one factor, 33.3% (4/12) with two factors, and 50.0% (1/2) with three concurrent factors. The Cochran–Armitage trend test confirmed a statistically significant linear trend (*p*-trend = 0.038). These data are presented in Table 3 and Figure 1.

### 3.6. Multivariate Logistic Regression

**Figure 2:**
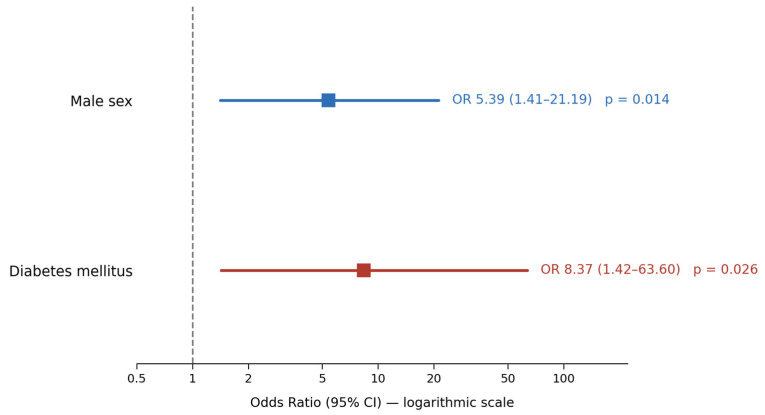
Forest plot of multivariate logistic regression. Squares represent odds ratios; horizontal bars represent 95% confidence intervals. Logarithmic x-axis. Vertical dashed line: OR 1 (no effect). OR: odds ratio; CI: confidence interval.

In the primary, parsimonious model (male sex and diabetes mellitus; events-per-variable = 12.5), both predictors remained independently associated with postoperative complications (male sex: OR 5.39, 95% CI 1.41–21.19, *p* = 0.014; diabetes mellitus: OR 8.37, 95% CI 1.42–63.60, *p* = 0.026). Results of the full covariate model, reported below and in Table 4, are presented as a pre-specified sensitivity analysis given its lower events-per-variable ratio (3.6). In the sensitivity (full-covariate) multivariate logistic regression model, two variables were independently and significantly associated with postoperative complications: male sex (OR 5.39; 95% CI 1.41–21.19; *p* = 0.014) and diabetes mellitus (OR 8.37; 95% CI 1.42–63.60; *p* = 0.026). Active smoking showed a numerically elevated OR (1.85; 95% CI 0.44–6.99; *p* = 0.377) that did not reach statistical significance, likely reflecting limited statistical power in this subgroup. Antithrombotic therapy was inversely associated with complications (OR 0.57; 95% CI 0.07–3.64; *p* = 0.569), though the wide confidence interval precludes meaningful interpretation. Age and deformity severity were not significantly associated with the occurrence of complications in multivariate analysis. The Hosmer–Lemeshow test indicated adequate model calibration (chi-squared [χ^2^] = 6.34; degrees of freedom [df] = 8; *p* = 0.61). Complete results are presented in Table 4 and Figure 2.

## 4. Discussion

The principal finding of this study is that, despite its overall functional efficacy, hallux valgus surgery cannot be regarded as a procedure that is neutral with respect to the patient’s systemic condition. A complication rate of 20.3% was observed in a consecutively recruited, unselected real-world cohort, and, crucially, the risk of adverse events increased in a statistically significant and clinically meaningful dose–response pattern with the accumulation of preoperative risk factors. These findings underscore the need for systematic preoperative risk factor assessment as an integral component of the surgical consultation, beyond the traditional focus on deformity severity and technical planning.

The overall complication rate of 20.3% is consistent with the mid-range of values reported in contemporary literature. Prior prospective and registry-based series have documented complication rates ranging from 8% to 35% depending on the osteotomy technique, follow-up duration, and definition of adverse events applied [9,10]. Recurrence was the most frequent adverse outcome in our series (12.2%), in agreement with published meta-analyses that have consistently identified deformity recurrence as the primary long-term complication following hallux valgus correction, with pooled rates between 4% and 16% [24,25,26]. It should be noted that the definition of recurrence adopted in this study (HVA > 20 degrees with symptomatic deformity at six months) may differ from those used in other reports, and this methodological heterogeneity should be considered when making cross-study comparisons.

Diabetes mellitus emerged as the factor with the strongest independent association with postoperative complications in multivariate analysis (OR 8.37; 95% CI 1.42–63.60; *p* = 0.026). This association is pathophysiologically coherent and consistent with the broader orthopedic literature. Hyperglycemia impairs the inflammatory and proliferative phases of wound healing, reduces neutrophil function, promotes peripheral microangiopathy, and alters collagen cross-linking-all of which represent mechanisms directly relevant to healing after osteotomy [16,18,19]. Large cohort studies of foot and ankle surgery have confirmed that diabetic patients carry substantially elevated rates of surgical site infection, hardware failure, and delayed union compared with nondiabetic controls, particularly in the presence of peripheral neuropathy or suboptimal glycemic control [18,19].

Two studies specifically focused on hallux valgus surgery reported more nuanced findings. Law et al. found no significant differences in complications or patient satisfaction between diabetic patients with adequate glycemic control (glycated hemoglobin, HbA1c < 8%) and nondiabetic controls undergoing osteotomy, suggesting that metabolic optimization may mitigate the excess risk [27]. Biz et al. similarly reported no significant difference in early complication rates following minimally invasive hallux valgus surgery in diabetic versus nondiabetic patients [28]. These results do not invalidate our findings; rather, they strengthen the clinical argument that the impact of diabetes on outcomes is not fixed but is modulated by glycemic control, neuropathic status, and preoperative patient selection. Our study cannot disaggregate these sub-effects due to the binary coding of diabetes status and the absence of HbA1c data, which represents a recognized limitation. Nevertheless, the magnitude of the observed OR supports implementing structured preoperative glycemic optimization protocols and enhanced postoperative surveillance in diabetic patients undergoing hallux valgus surgery.

Active smoking showed a clinically relevant but statistically non-significant trend toward increased complication risk (OR 1.85; *p* = 0.377). This finding should not be interpreted as evidence of the absence of an effect. Smoking impairs wound healing through well-documented mechanisms including peripheral vasoconstriction, carboxyhemoglobin-mediated reduction in tissue oxygen delivery, inhibition of fibroblast proliferation, and immune suppression [17,20]. Both Beahrs et al. and Pour Jafar et al. have synthesized evidence from the foot and ankle surgery literature confirming these deleterious effects [17,20], and the numerically elevated OR in our series is directionally consistent with these reports. The lack of statistical significance is most likely attributable to insufficient statistical power given the available sample size, and to the binary coding of smoking status, which cannot capture the dose-dependent effects related to pack-years of exposure or time since cessation. Smoking cessation counseling at least six weeks before elective foot surgery remains a best-practice recommendation, and our data provide additional directional support for this approach.

The association between male sex and complications (OR 5.39; 95% CI 1.41–21.19) warrants cautious interpretation. Males represented only 17.9% of the cohort (*n* = 22), resulting in wide confidence intervals that substantially limit the precision of this estimate. From a clinical perspective, hallux valgus in male patients has been associated with a potentially distinct biomechanical and morphological profile, including a higher prevalence of metatarsus primus varus and a different pattern of etiological factors compared with female patients [6]. Whether this translates into genuinely higher complication risk or whether the observed association reflects residual confounding by unrecorded comorbidities or biomechanical characteristics that were not captured in the analysis cannot be determined from the current data. This observation should be treated as a hypothesis warranting confirmation in future prospective studies with larger male representation.

Given the limited number of events (*n* = 25), the primary multivariate model was restricted to two predictors identified through bivariate significance and established prior evidence—male sex and diabetes mellitus—yielding 12.5 events per variable (EPV), consistent with conventional recommendations for model stability. The broader model including all candidate covariates (age, sex, active smoking, diabetes mellitus, antithrombotic therapy, and deformity severity), shown in Table 4, is reported as a pre-specified secondary/sensitivity analysis rather than as confirmatory inference. The EPV ≥ 10 rule is a widely used heuristic rather than a strict statistical requirement; simulation studies have shown that logistic models with EPV as low as 5–9 can still yield directionally reliable estimates provided multicollinearity is low and calibration is adequate, both of which were verified here (variance inflation factors < 5 for all covariates; Hosmer–Lemeshow χ^2^ = 6.34, df = 8, *p* = 0.61) [29,30]. The correspondingly wide confidence intervals for some covariates in the sensitivity model (e.g., diabetes mellitus, 95% CI 1.42–63.60) reflect this reduced precision and should be interpreted as exploratory pending confirmation in larger, prospective, multicenter cohorts.

The dose–response relationship observed with the accumulated risk factor index (15.9% to 50.0% complication rate across the 0–3 score range; *p*-trend = 0.038; exact 95% CIs 7.9–27.3% and 1.3–98.7% for the 0- and 3-factor strata, respectively, reflecting the markedly lower precision of the latter) is arguably the most immediately applicable clinical finding of this study. The progressive and significant nature of this trend demonstrates that co-occurrence of risk factors confers an additive burden that goes beyond the individual effects of each condition. This is consistent with the general principle underlying cumulative comorbidity burden tools such as the Charlson Comorbidity Index [21], which have demonstrated across multiple surgical contexts that the simultaneous presence of multiple systemic conditions amplifies perioperative risk in a nonlinear fashion. The small sizes of the high-risk-factor-burden subgroups (*n* = 12 for two factors; *n* = 2 for three factors) limit the precision of the higher point estimates and require confirmation in larger series, but the overall trend is statistically robust and clinically interpretable. From a practical standpoint, the accumulated risk factor index is intended as a simple bedside stratification tool rather than a precisely calibrated risk score; its clinical value lies in flagging patients with two or more risk factors for more thorough preoperative assessment, explicit risk counseling, and closer postoperative surveillance, pending confirmation of the exact magnitude of risk in larger prospective cohorts.

This study has several limitations that should be considered when interpreting its findings. First, the retrospective design precludes causal inference and entails the inherent risk of incomplete or inconsistent data recording. Second, key metabolic variables including glycated hemoglobin, fasting glucose levels at the time of surgery, ankle-brachial index, and smoking pack-years were not available in the clinical records and could not be incorporated into the analysis. Third, the small sizes of the high-risk-factor-burden subgroups limit the statistical precision of the subgroup-level estimates; exact confidence intervals are reported in Table 2 to make this precision transparent. Fourth, a standardized surgical complication classification system such as the Clavien–Dindo scale was not applied, which may limit comparability with future studies. Fifth, the single-center design restricts generalizability, and referral patterns specific to the Hospital Rafael Mendez catchment area may not be representative of other clinical settings. Sixth, the analysis was restricted to three retrospectively ascertainable risk factors (active smoking, diabetes mellitus, antithrombotic therapy) rather than a full comorbidity and medication profile; antithrombotic therapy in particular should be understood as a proxy for underlying cardiovascular or thromboembolic disease rather than an independent risk factor. Seventh, given the limited number of events, the full-covariate regression model should be interpreted as an exploratory sensitivity analysis rather than a confirmatory model; the parsimonious primary model is less susceptible to overfitting but was necessarily limited in scope. Future prospective, multicenter studies incorporating objective metabolic markers, a complete comorbidity and medication profile, standardized complication classification systems, and longer follow-up periods are needed to build on these findings.

## 5. Conclusions

Hallux valgus surgery was associated with clinically relevant improvement in pain and function at 12-month follow-up, as reflected by better VAS pain and AOFAS-MI functional scores; given the retrospective design of the study, these findings should be interpreted as associations rather than causal effects. Preoperative risk factors significantly modulate the risk of postoperative complications, and their accumulation follows a dose–response pattern that is both statistically significant and, pending confirmation in larger prospective studies, potentially clinically actionable.

In multivariate analysis, diabetes mellitus and male sex were independently and significantly associated with a higher incidence of complications in the sensitivity model, a hypothesis-generating finding requiring confirmation in adequately powered prospective studies, while the progressive accumulation of risk factors was associated with a near-linear increase in complication rates, from 15.9% with no risk factors to 50.0% with three concurrent conditions (though the latter estimate is imprecise, exact 95% CI 1.3–98.7%, given *n* = 2). These findings support the systematic integration of preoperative risk factor assessment into the surgical consultation for hallux valgus, alongside the conventional evaluation of deformity severity.

Practical implications may include metabolic optimization prior to surgery in patients with diabetes mellitus (targeting HbA1c < 7.5% when feasible). Similarly, structured smoking cessation counseling at least six weeks before elective surgery and individualized postoperative follow-up intensity calibrated to the patient’s accumulated risk factor burden may also be included. These implications should be regarded as clinically reasonable interpretations of the observed associations rather than definitive recommendations derived from causal evidence. Patients with two or more of the identified risk factors may represent a subgroup with increased postoperative risk and may therefore benefit from more explicit preoperative counseling about the elevated complication probability, greater intraoperative caution, and closer postoperative follow-up. Even so, larger prospective studies are needed to confirm the magnitude and consistency of these associations and to determine their usefulness for clinical decision-making.

## Figures and Tables

**Table 1:** Baseline characteristics of the sample (*n* = 123).

Variable	Result
Patients included	123
Mean age, years (SD)	57.4 (12.3)
Range of age, years	22–81
Women, *n* (%)	101 (82.1%)
Mean BMI, kg/m^2^ (SD)	27.3 (4.1)
Active smokers, *n* (%)	39 (31.7%)
Diabetes mellitus, *n* (%)	18 (14.6%)
Antiplatelet/anticoagulant therapy, *n* (%)	16 (13.0%)
Mild hallux valgus, *n* (%)	28 (22.8%)
Moderate hallux valgus, *n* (%)	73 (59.3%)
Severe hallux valgus, *n* (%)	22 (17.9%)
Procedures by Surgeon A, *n* (%)	72 (58.5%)
Procedures by Surgeon B, *n* (%)	51 (41.5%)
Mean follow-up, months (range)	12 (10–18)
Preoperative VAS score, mean (SD)	7.4 (1.1)
Postoperative VAS score, mean (SD)	1.2 (0.7)
Preoperative AOFAS-MI score, mean (SD)	56.0 (7.5)
Postoperative AOFAS-MI score, mean (SD)	91.0 (4.7)

BMI: body mass index; SD: standard deviation; VAS: Visual Analogue Scale; AOFAS-MI: American Orthopaedic Foot and Ankle Society Hallux Metatarsophalangeal-Interphalangeal scale.

**Table 2:** Complication rate by individual risk factor.

Risk Factor	Present, *n* (%)	Absent, *n* (%)	*p*-Value
Active smoking	39 (28.2)	84 (15.5)	0.120
Diabetes mellitus	18 (38.9)	105 (17.1)	0.049
Antithrombotic therapy	16 (31.3)	107 (18.7)	0.310

Rates calculated using the chi-squared test or Fisher’s exact test, as appropriate.

**Table 3:** Risk factor burden and complication rate.

No. of Risk Factors	*n*	% of Total	Patients with Complications, *n*	Complication Rate, %
0	63	51.2	10	15.9
1	46	37.4	10	21.7
2	12	9.8	4	33.3
3	2	1.6	1	50
Total	123	100	25	20.3

Risk factors: active smoking, diabetes mellitus, and antiplatelet or anticoagulant therapy. Cochran–Armitage trend test: *p*-trend = 0.038. Exact (Clopper–Pearson) 95% confidence intervals are reported in the Discussion.

**Table 4:** Multivariate logistic regression: risk factors associated with postoperative complications (*n* = 123).

Variable	OR	95% CI	*p*-Value	Sig.
Age (per year)	1.03	0.99–1.08	0.197	
Male sex	5.39	1.41–21.19	0.014	*
Active smoking	1.85	0.44–6.99	0.377	
Diabetes mellitus	8.37	1.42–63.60	0.026	*
Antiplatelet/anticoagulant therapy	0.57	0.07–3.64	0.569	
Moderate hallux valgus (ref.: mild)	0.82	0.22–3.44	0.775	
Severe hallux valgus (ref.: mild)	0.58	0.08–3.51	0.557	

OR: odds ratio; CI: confidence interval; Sig.: significance. * *p* < 0.05. Reference for deformity severity: mild hallux valgus. Hosmer–Lemeshow goodness-of-fit test: χ^2^ = 6.34, df = 8, *p* = 0.61.

## Data Availability

The data presented in this study are available on reasonable request from the corresponding author. The data are not publicly available due to privacy and ethical restrictions concerning patient data.
